# Pairwise Multiple Comparison Adjustment Procedure for Survival Functions with Right-Censored Data

**DOI:** 10.1155/2017/9270450

**Published:** 2017-10-12

**Authors:** Ertugrul Colak, Hulya Ozen, Busra Emir, Setenay Oner

**Affiliations:** Department of Biostatistics, Faculty of Medicine, Eskisehir Osmangazi University, Eskisehir, Turkey

## Abstract

The aim of this study is to propose a new pairwise multiple comparison adjustment procedure based on Genz's numerical computation of probabilities from a multivariate normal distribution. This method is applied to the results of two-sample log-rank and weighted log-rank statistics where the survival data contained right-censored observations. We conducted Monte Carlo simulation studies not only to evaluate the familywise error rate and power of the proposed procedure but also to compare the procedure with conventional methods. The proposed method is also applied to the data set consisting of 815 patients on a liver transplant waiting list from 1990 to 1999. It was found that the proposed method can control the type I error rate, and it yielded similar power as Tukey's and high power with respect to the other adjustment procedures. In addition to having a straightforward formula, it is easy to implement.

## 1. Introduction

Survival analysis is based on making inferences from the time-to-event data. It provides many statistical procedures for studying the data, including the time from a correctly identified origin until the occurrence of a certain event [1]. One of the main interests in survival analysis is evaluating the equality of survival functions for different groups. Many tests such as log-rank and weighed log-rank have been proposed [2–8]. Although these tests made important contributions to survival analysis, they can only provide overall or two-sample comparison results. Researchers will fail if they use these tests to compare one with another in a multigroup study design because the probability of making at least one type I error will be increased above the critical level. To prevent this mistake, pairwise multiple comparison procedures are needed. In case of the inequality of more than two groups, it is necessary to correctly decide which groups are different from the others. The appropriate way to control the type I error is to consider the familywise error (FWE) rate, which is the probability of making at least one type I error when making all pairwise comparisons [9].

Adjustment methods such as Bonferroni, Holm, and Sidak methods are commonly used in the literature. However, in survival analysis this topic has only recently been studied. Adjustment methods are applied to the results of two-sample log-rank and weighted log-rank tests. Bonferroni is the most preferred method among the others. In a two-sided test, Bonferroni assumes the significance level as (*α*/2) × *m*, where *m* is the number of pairwise comparisons in the study, but it fails when controlling the familywise error rate. In spite of its simplicity, it has been determined to be a conservative method in survival analysis [9, 10]. Logan et al. proposed two different adjustment methods that consider the correlation among the pairwise tests [9]. One of the methods was derived from multivariate normal distribution, while the other was obtained from a simulated martingales approach. Koziol and Reid used the Sidak adjustment method to calculate the pairwise comparisons results of weighted log-rank tests. Although it generates more consistent results than Bonferroni's, it was also found to be conservative [11]. Not only were pairwise multiple comparisons proposed, but comparisons against a single control group were also proposed for survival functions with right-censored data in the statistical literature. Chakraborti and Desu developed linear rank tests, and Chen proposed a generalized Steel's test and an alternative method to the generalization of Steel's test [12–15].

The aim of this study is to propose a new pairwise multiple comparison adjustment procedure based on Genz's numerical computation of probabilities from a multivariate normal distribution [16, 17]. This method is applied to the results of two-sample log-rank and weighted log-rank statistics where the survival data contained right-censored observations. In Section 2, some notations are given, and the construction of the simulation study is detailed. In the simulation studies SAS PROC LIFETEST and R package with mvtnorm library were used. Moreover, all adjustment methods are applied to a real life-time data set and they are compared with each other. The results and discussion about other studies are evaluated in Section 3. Finally, conclusions are mentioned in Section 4.

## 2. Materials and Methods

### 2.1. Notation and Background

Let *S*_*k*_(*t*) be the survival function of the *k*th group for  *k* = 1,…, *K*, where *K* is the number of groups. The null and alternative hypotheses for the survival functions are

(1) H0: S1t=⋯=SKtH1:  at least one of the Skt's is different for some t≤τ,

where *τ* is the largest observed time.

Let (*T*_*i*_, *δ*_*i*_, *X*_*i*_, *w*_*i*_), for *i* = 1,…, *n*, indicates that an independent sample for right-censored survival data where *T*_*i*_ is right-censored time, *δ*_*i*_ is the indicator variable for censoring (*δ*_*i*_ = 0 if *T*_*i*_ is censored; *δ*_*i*_ = 1 if *T*_*i*_ is an event time), *X*_*i*_ is the group indicator of 1,…, *K*, and *w*_*i*_ is a weight function. Let *t*_1_ < *t*_2_ < ⋯<*t*_*D*_  *j* = 1,…, *D* be distinct event times in the sample. At time *t*_*j*_, for the *k*th group, let *Y*_*jk*_ = ∑_*i*:*T*≥*t*_*j*__ *I*(*X*_*i*_ = *k*) and *d*_*jk*_ = ∑_*i*:*T*_*i*_=*t*_*j*__ *δ*_*i*_ *I*(*X*_*i*_ = *k*) denote the number of individuals at risk and the number of events, respectively. Let *Y*_*j*_ = ∑_*k*=1_^*K*^ *Y*_*jk*_ and *d*_*j*_ = ∑_*k*=1_^*K*^ *d*_*jk*_ denote the number individuals at risk and the number of events, respectively. The weighted number of individuals at risk in the *k*th group is *Y*_*jk*_^*w*^ = ∑_*i*:*T*_*i*_≥*t*_*j*__ *w*_*i*_ *I*(*X*_*i*_ = *k*), while the weighted number of events in the *k*th group is *d*_*jk*_^*w*^ = ∑_*i*:*T*_*i*_=*t*_*j*__ *w*_*i*_ *δ*_*i*_ *I*(*X*_*i*_ = *k*). Let *Y*_*j*_^*w*^ = ∑_*k*=1_^*K*^ *Y*_*jk*_^*w*^ and *d*_*j*_^*w*^ = ∑_*k*=1_^*K*^ *d*_*jk*_^*w*^ indicate the weighted number of individuals at risk and the weighted number of events, respectively.

For testing the null hypothesis, the test statistics have the form of a *K*-vector **R** = (*r*_1_, *r*_2_,…,*r*_*k*_)′, where

(2) $$\begin{array}{c} r_{k}=\overset{D}{\underset{j=1}{\sum }}\left(\;d_{jk}^{w}-Y_{jk}^{w}\frac{d_{j}^{w}}{Y_{j}^{w}}\;\right). \end{array}$$

Variance of *r*_*k*_ and covariance for *r*_*k*_ and *r*_*h*_ are as follows, respectively:

(3) vkk=∑j=1DdjYj−djYjYj−1∑i=1YjYjkwYjw2wi2IXi≠k+Yjw−YjkwYjw2wi2IXi=k

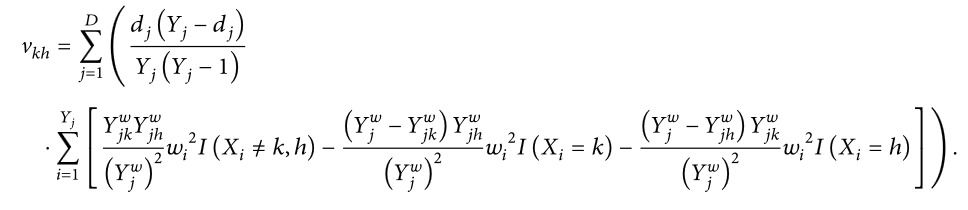
(4)
Because the sum of *r*_*k*_ is equal to 0, they are linearly dependent. Accordingly, the general test statistic is constructed by selecting any *K* − 1 of *r*_*k*_'s. The test statistic, (*r*_1_, *r*_2_,…, *r*_*K*−1_)**V**_(*K* − 1)×(*K* − 1)_^−1^(*r*_1_, *r*_2_,…,*r*_*K*−1_)′, follows a Chi-square distribution with *K* − 1 degrees of freedom, where **V** is the variance-covariance matrix.

Let *m* be the number of all pairwise comparisons where  *m* = *K*(*K* − 1)/2. The two-sided test statistic, *Z*_*kh*_, compares the groups *k* and *h* and follows a standard normal distribution.

(5) $$\begin{array}{c} Z_{kh}=\frac{r_{k}-r_{h}}{\sqrt{v_{kk}+v_{hh}-2v_{kh}}}. \end{array}$$

The unadjusted *p* value is  *p*_*kh*_ = *P*(*χ*_1_^2^ > *Z*_*kh*_^2^). The multiple comparison procedures that are used to adjust the *p* values in this study are shown below:

Bonferroni: *p*_*kh*_ = min⁡[1, *m* × *P*(*χ*_1_^2^ > *Z*_*kh*_^2^)].
Scheffé: *p*_*kh*_ = *P*(*χ*_*K*−1_^2^ > *Z*_*kh*_^2^).
Sidak: *p*_*kh*_ = 1 − [1 − *P*(*χ*_1_^2^ > *Z*_*kh*_^2^)]^*m*^.
Studentized Maximum Modulus: *p*_*kh*_ = 1 − [2 × Φ(*Z*_*kh*_)]^*m*^.
Tukey: $p_{kh}=1-\int_{-\infty }^{\infty }K\phi \left(u\right){\left[\Phi \left(u\right)-\Phi \left(u-\sqrt{2}Z_{kh}\right)\right]}^{K-1}du$,

where *ϕ* and Φ are standard normal and cumulative standard normal functions, respectively.

### 2.2. Proposed Adjustment Procedure

**Z** = [*Z*_12_,…,*Z*_1*K*_, *Z*_23_,…,*Z*_2*K*_,…*Z*_*K*−1,*K*_]_*m*_ has a multivariate normal distribution with a mean of zero and a variance-covariance matrix Σ. Under the null hypothesis, the elements of Σ follow a rule which is Cov(*Z*_*kh*_, *Z*_*kh*′_) = 0.5, Cov(*Z*_*kh*_, *Z*_*k*′*h*′_) = 0, and Cov(*Z*_*kh*_, *Z*_*hk*′_) = −0.5, where 1 ≤ *k* ≠ *h* ≠ *k*′ ≠ *h*′ ≤ *K* [9, 14, 15].

The function of (**a**, **b**, Σ) is

(6) ΦZ ∣ a,b,Σ=1Σ2πm∫a1b1∫a2b2⋯∫ambme−1/2Z′Σ−1Zdz,−∞≤ai≤bi≤∞,  i=1,…,m.

For the integration shown above, we used “mvtnorm” library, released February 2, 2016, for numerical computation in R program. There are three algorithms available for evaluating normal probabilities: The default is the randomized Quasi-Monte-Carlo procedure by Genz (1992, 1993). We used this approach because it is easy to use and calculate with R program.

The proposed multiple adjustment procedures for the pairwise comparison of the *k*th and *h*th groups are obtained using Φ and shown below:

(7) $$\begin{array}{c} p_{kh}=\min\left[\;1,2\times P\left(\;1-\Phi \left(\;Z\;|\;a,b,\Sigma \;\right)\;\right)\;\right], \end{array}$$

where **a** = [−*∞*,…,−*∞*]_*m*_ and **b** = [*Z*_*kh*_,…,*Z*_*kh*_]_*m*_.

Additionally, the critical value for the pairwise comparison can be evaluated with

(8) $$\begin{array}{c} Z_{c}=\Phi^{-1}\left(\;1-\frac{\alpha }{2}\;\right). \end{array}$$

### 2.3. Simulation Study

We performed Monte Carlo simulation studies to examine the proposed and conventional adjustment procedures. The FWE rate and power of the adjustment procedures were obtained through the simulation results. In this study, the number of groups was determined as *K* = 4; *X*_*i*_ = 1,…, 4. The sample sizes were considered equal for each group as *n* = 50, 150, and 250 to estimate the FWE rate, while it was just 250 in the power study. The right-censored survival times *T*_*i*_ were derived from the exponential *T*_*i*_ ~ exp⁡(*λ*_*X*_*i*__) and lognormal distribution *T*_*i*_ ~ lognormal(*μ*_*X*_*i*__, *σ* ^2^). The censoring rate was considered to be 30%. Therefore, the censoring variable was generated from a Bernoulli distribution *δ*_*i*_ ~ Bernoulli(*p* = 0.70). Note that the censoring rate was fixed for each group in the FWE rate and power study. To obtain the adjusted *p* values, Bonferroni, Scheffé, Sidak, SMM, Tukey, and the proposed adjustment procedure were applied to the pairwise comparison results of log-rank and weighted log-rank tests. For each scenario 1000 data sets were simulated independently.

To compare the FWE rates of the adjustment procedures, the survival times for each group were generated from the standard exponential distribution with *λ*_*k*_ = 1 and the lognormal distribution with a mean of *μ*_*k*_ = 0 and scale parameter  *σ*_*k*_ = 0.5. The estimated FWE rates of the adjustment procedures were evaluated with respect to the critical value *α* = 0.05. In the power study, we used exponential distributions with various parameters *λ*_*k*_ and lognormal distributions with *σ*_*k*_ = 0.5 but different values of *μ*_*k*_. For power calculation, we calculated the probability of making a correct decision only for unequal pairs. Note that the exponential distribution provides a proportional hazards model while the lognormal distribution corresponds to location shifts in log survival times. The lognormal distributions with various means were used because they have different hazards at early times [15].

### 2.4. Application Data

The data set was obtained from the free data sets used in the R package, “survival” [18, 19]. It consisted of 815 patients on a liver transplant waiting list from 1990 to 1999 with six variables: age at the addition to the waiting list, sex, blood type, year in which a patient entered the waiting list, and time from the entry to end point. The final disposition of the patients was categorized as received a transplant, died while waiting, withdrew from the list, or censored. Blood type is a crucial factor which affects the waiting time for transplantation. Although the liver donation from subjects with blood type O can be used by patients with all blood types, a patient with blood type O can only receive donation from the subjects with blood type O. Thus, patients with O blood type on the waiting list have a disadvantage. These data is of historical interest and provides a useful example of competing risks, but it has little relevance to current practice. We used these data as an example to demonstrate the comparison of the proposed and conventional adjustment techniques on a real data set. We considered that the event is receiving a transplant, while the other categories of final disposition are censored.

## 3. Results and Discussion

Table 1 shows the simulation results for the estimated FWE rates of the proposed and conventional adjustment procedures for exponential survival distribution with different sample sizes. Under the null hypothesis, FWE rates are expected to be 0.05. As the sample size increases, estimates get closer to the targeted value in all adjustment procedures. It is obvious that the Scheffé method is the most inefficient among the others. The proposed adjustment procedure and Tukey's present similar results. It can be seen that both adjustment procedures can control the type I error even for small samples. Their performance is followed by Sidak, SMM, and Bonferroni procedures. In Table 2, the estimates of the FWE rates for the survival times from the lognormal distribution with the parameters *μ*_*k*_ = 0 and *σ*_*k*_ = 0.5 are given. Unlike the previous simulation results, not all procedures give estimates that are close to the targeted value. The proposed adjustment procedure and Tukey's provide the most efficient results. The decrease in the performance of the adjustment procedures could depend on the type of distributions. Because an exponential distribution provides a more appropriate proportional hazard model than a lognormal distribution, this affects the performance of the log-rank and the weighted log-rank tests. Therefore, the adjustment procedures tend to cause errors.

Next, the simulation results are calculated for the power of the proposed and conventional adjustment procedures for the exponential survival distribution. Under a variety of hypothesis configurations denoted by *λ*_*k*_, the estimated power results are given in Table 3. As the values of *λ*_*k*_ become different from each other, the power of all of the adjustment procedures decreases rapidly. The proposed adjustment procedure and Tukey's provide similar results with the highest power. We also conducted additional simulations where the survival times were derived from a lognormal distribution. The estimates of power under alternative configurations of *μ*_*k*_ are given in Table 4. Inefficient power results are only seen when all of the *μ*_*k*_ values are different. Moreover, the performance of all of the adjustment procedures gives very similar results. In all the simulation results, it can be seen that there is no notable difference between the log-rank and weighted log-rank tests.

**Figure 1:**
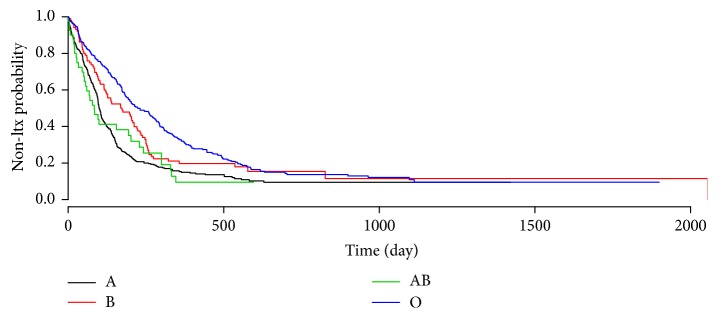
Kaplan-Meier estimates of not receiving a transplant for each blood type group.

Descriptive statistics of the application data set are given in Table 5 and the survival functions of the groups are shown in Figure 1. The overall comparison of blood type groups is conducted with log-rank test. The result is found to be highly significant (*χ* ^2^ = 45.5, df = 3, and *p* < 0.001). Thus, pairwise comparisons followed by multiple adjustment procedures were conducted, and the results are given in Table 6. All of the adjustment procedures had the same conclusions and present results that are similar to those that we observed in the simulation studies. The comparison results show that, with the exception of the pair of B and AB, all of the blood types are highly different from each other. The *p* values obtained for each comparative test for the application data showed significant differences (*p* < 0.001) between the survival times of the blood groups except for the comparison of AB and B groups (*p* > 0.05). The results can be seen in Kaplan-Meier curves represented in Figure 1. The survival curves show a proportional structure until the middle of the 0–500-day interval. Also, the survival curves of AB and B blood groups are closer to each other compared to the other groups.

A statistician can use this method in usual data analysis procedure as follows. For example, to calculate the adjusted *p* value for the comparison of the groups *k* and *h*,

1. calculate *Z*_*kh*_ and Σ defined in Section 2.1,
2. use pvnorm command in mvtnorm library in R as follows:
3. *l* = rep(−Inf, *m*).
4. *u* = rep(*Z*_*kh*_, *m*).
5. *a* = pmvnorm(lower = *l*, upper = *u*, mean = 0, corr = sigma).
6. *p* = 2*∗*(1 − (*a*[1] + attributes(*a*)$error)),
7. where *m* is the number of all comparisons, and sigma is Σ.

## 4. Conclusions

In this study, we proposed a multiple adjustment procedure for the pairwise comparisons of survival functions with right-censored data. We conducted Monte Carlo simulation studies not only to evaluate the FWE rate and power of the proposed procedure but also to compare the procedure with conventional methods. It was found that the proposed method can control the type I error rate, and it yielded similar power as Tukey's and high power with respect to the other adjustment procedures. In addition to having a straightforward formula, it is easy to implement.

This study has some limitations. The main issue was that the simulations were performed by using proposed and conventional methods. However, comparisons can be extended including the methods such as that of Logan et al. (2005) in the comparison. Logan et al. proposed two different adjustment methods that consider the correlation among the pairwise tests. One of the methods was derived from multivariate normal distribution, while the other was obtained from a simulated martingales approach. These models may work well for the data with proportional hazard structure. Future researches should take into account the models for comparisons.

## Figures and Tables

**Table 1:** FWE rates of the proposed and conventional adjustment procedures for *K* = 4 and *α* = 0.05 and exponential survival distribution with *λ*_*k*_ = 1.

Sample size	Tests	Proposed and conventional adjustment techniques						
		Unadjusted	Bonferroni	Scheffé	Sidak	SMM	Tukey	Proposed
50	Fleming	0.194	0.039	0.031	0.040	0.040	0.053	0.053
	Log-rank	0.187	0.040	0.024	0.040	0.040	0.046	0.046
	ModPeto	0.196	0.040	0.031	0.043	0.043	0.054	0.054
	Peto	0.194	0.039	0.031	0.042	0.042	0.054	0.054
	Tarone	0.193	0.041	0.027	0.041	0.041	0.053	0.053
	Wilcoxon	0.204	0.043	0.029	0.044	0.044	0.052	0.052

150	Fleming	0.206	0.034	0.022	0.035	0.035	0.039	0.039
	Log-rank	0.185	0.036	0.019	0.037	0.037	0.044	0.044
	ModPeto	0.204	0.033	0.023	0.034	0.034	0.038	0.038
	Peto	0.206	0.034	0.022	0.034	0.034	0.038	0.038
	Tarone	0.198	0.035	0.020	0.035	0.035	0.045	0.045
	Wilcoxon	0.211	0.038	0.023	0.038	0.038	0.045	0.045

250	Fleming	0.214	0.045	0.032	0.046	0.046	0.057	0.057
	Log-rank	0.209	0.043	0.030	0.044	0.044	0.049	0.049
	ModPeto	0.214	0.045	0.032	0.046	0.046	0.057	0.057
	Peto	0.214	0.045	0.032	0.046	0.046	0.057	0.057
	Tarone	0.210	0.047	0.033	0.047	0.047	0.054	0.054
	Wilcoxon	0.209	0.044	0.029	0.045	0.045	0.056	0.056

**Table 2:** FWE rates of the proposed and conventional adjustment procedures for *K* = 4 and *α* = 0.05 and log-normal survival distribution with *μ*_*k*_ = 0 and *σ*_*k*_ = 0.5.

Sample size	Tests	Proposed and conventional adjustment techniques						
		Unadjusted	Bonferroni	Scheffé	Sidak	SMM	Tukey	Proposed
50	Fleming	0.188	0.035	0.023	0.035	0.035	0.041	0.041
	Log-rank	0.199	0.038	0.021	0.038	0.038	0.043	0.043
	ModPeto	0.187	0.036	0.023	0.036	0.036	0.041	0.041
	Peto	0.189	0.035	0.023	0.036	0.036	0.041	0.041
	Tarone	0.182	0.032	0.022	0.033	0.033	0.041	0.041
	Wilcoxon	0.182	0.038	0.019	0.038	0.038	0.047	0.047

150	Fleming	0.202	0.046	0.025	0.046	0.046	0.051	0.051
	Log-rank	0.220	0.043	0.030	0.043	0.043	0.050	0.050
	ModPeto	0.200	0.045	0.025	0.046	0.046	0.051	0.051
	Peto	0.201	0.045	0.025	0.046	0.046	0.051	0.051
	Tarone	0.210	0.041	0.029	0.043	0.043	0.051	0.051
	Wilcoxon	0.196	0.044	0.023	0.044	0.044	0.051	0.051

250	Fleming	0.196	0.040	0.024	0.041	0.041	0.049	0.049
	Log-rank	0.201	0.037	0.023	0.037	0.037	0.044	0.044
	ModPeto	0.197	0.040	0.024	0.040	0.040	0.049	0.049
	Peto	0.196	0.040	0.024	0.041	0.041	0.049	0.049
	Tarone	0.195	0.045	0.023	0.046	0.046	0.049	0.049
	Wilcoxon	0.202	0.032	0.021	0.034	0.034	0.042	0.042

**Table 3:** Power of the proposed and conventional adjustment procedures for *K* = 4 and *α* = 0.05,and exponential survival distribution with different *λ*_*k*_.

Parameters	Tests	Proposed and conventional adjustment techniques						
(*λ*_1_, *λ*_2_, *λ*_3_, *λ*_4_)		Unadjusted	Bonferroni	Scheffé	Sidak	SMM	Tukey	Proposed
(2.25, 1.50, 1.50, 1.50)	Fleming	0.765	0.594	0.523	0.597	0.597	0.618	0.618
	Log-rank	0.858	0.785	0.726	0.784	0.784	0.802	0.802
	ModPeto	0.763	0.593	0.521	0.593	0.593	0.618	0.618
	Peto	0.764	0.593	0.522	0.597	0.597	0.618	0.618
	Tarone	0.789	0.654	0.595	0.657	0.657	0.676	0.676
	Wilcoxon	0.725	0.506	0.428	0.507	0.507	0.535	0.535

(2.25, 2.25, 1.50, 1.50)	Fleming	0.757	0.514	0.437	0.516	0.516	0.541	0.541
	Log-rank	0.823	0.627	0.555	0.629	0.629	0.665	0.665
	ModPeto	0.756	0.512	0.436	0.516	0.516	0.539	0.539
	Peto	0.757	0.513	0.436	0.516	0.516	0.541	0.541
	Tarone	0.789	0.558	0.490	0.560	0.560	0.588	0.588
	Wilcoxon	0.713	0.442	0.358	0.447	0.447	0.468	0.468

(2.25, 1.75, 1.75, 1.25)	Fleming	0.243	0.032	0.017	0.033	0.033	0.045	0.045
	Log-rank	0.368	0.063	0.034	0.064	0.064	0.080	0.080
	ModPeto	0.243	0.032	0.017	0.033	0.033	0.044	0.044
	Peto	0.243	0.032	0.017	0.033	0.033	0.044	0.044
	Tarone	0.290	0.046	0.024	0.047	0.047	0.055	0.055
	Wilcoxon	0.186	0.026	0.009	0.026	0.026	0.031	0.031

(2.50, 2.00, 1.50, 1.00)	Fleming	0.168	0.010	0.002	0.010	0.010	0.014	0.014
	Log-rank	0.269	0.023	0.006	0.024	0.024	0.035	0.035
	ModPeto	0.167	0.009	0.002	0.010	0.010	0.013	0.013
	Peto	0.167	0.010	0.002	0.010	0.010	0.014	0.014
	Tarone	0.204	0.012	0.006	0.013	0.013	0.018	0.018
	Wilcoxon	0.121	0.005	0.000	0.005	0.005	0.005	0.005

**Table 4:** Power of the proposed and conventional adjustment procedures for *K* = 4 and *α* = 0.05 and log-normal survival distribution with different *μ*_*k*_ and *σ*_*k*_ = 0.5.

Parameters	Tests	Proposed and conventional adjustment techniques						
(*μ*_1_, *μ*_2_, *μ*_3_, *μ*_4_)		Unadjusted	Bonferroni	Scheffé	Sidak	SMM	Tukey	Proposed
(0.5, 0, 0, 0)	Fleming	0.871	0.978	0.985	0.978	0.978	0.976	0.976
	Log-rank	0.901	0.995	0.996	0.995	0.995	0.991	0.991
	ModPeto	0.871	0.978	0.985	0.978	0.978	0.976	0.976
	Peto	0.871	0.978	0.985	0.978	0.978	0.976	0.976
	Tarone	0.880	0.979	0.989	0.978	0.978	0.973	0.973
	Wilcoxon	0.869	0.978	0.985	0.977	0.977	0.973	0.973

(0.5, 0.5, 0, 0)	Fleming	0.922	0.987	0.992	0.987	0.987	0.984	0.984
	Log-rank	0.923	0.988	0.995	0.988	0.988	0.985	0.985
	ModPeto	0.923	0.987	0.992	0.987	0.987	0.984	0.984
	Peto	0.923	0.987	0.992	0.987	0.987	0.984	0.984
	Tarone	0.928	0.987	0.994	0.987	0.987	0.986	0.986
	Wilcoxon	0.931	0.986	0.992	0.986	0.986	0.983	0.983

(0.3, 0, 0, −0.3)	Fleming	0.962	0.982	0.980	0.982	0.982	0.984	0.984
	Log-rank	0.947	0.940	0.906	0.940	0.940	0.949	0.949
	ModPeto	0.962	0.982	0.980	0.982	0.982	0.984	0.984
	Peto	0.962	0.982	0.980	0.982	0.982	0.984	0.984
	Tarone	0.958	0.979	0.974	0.979	0.979	0.980	0.980
	Wilcoxon	0.962	0.985	0.979	0.985	0.985	0.984	0.984

(0.5, 0.3, −0.3, −0.5)	Fleming	0.716	0.332	0.245	0.336	0.336	0.373	0.373
	Log-rank	0.551	0.260	0.199	0.262	0.262	0.293	0.293
	ModPeto	0.716	0.329	0.244	0.337	0.337	0.371	0.371
	Peto	0.716	0.332	0.245	0.337	0.337	0.371	0.371
	Tarone	0.712	0.345	0.278	0.347	0.347	0.395	0.395
	Wilcoxon	0.698	0.266	0.191	0.271	0.271	0.304	0.304

**Table 5:** Descriptive statistics for the liver transplant waiting list data.

Blood groups	LTX	Censored	Total	Percent censored	Median follow-up (days)	95% confidence interval	
						Lower	Upper
A	269	56	325	0.172	100	95	108
AB	33	8	41	0.195	84	52	202
B	78	25	103	0.243	173	116	212
0	256	90	346	0.260	223	193	276
Total	636	179	815	0.219			

**Table 6:** Test statistics and the adjusted *p* values of the proposed and conventional adjustment techniques for the liver transplant waiting list data.

Tests	Blood groups		Test statistics	Proposed and conventional adjustment techniques						
			|*Z*|	Unadjusted	Bonferroni	Scheffé	Sidak	SMM	Tukey	Proposed
Fleming	A	AB	5.106	<0.0001	<0.0001	<0.0001	<0.0001	<0.0001	<0.0001	<0.0001
	A	B	5.236	<0.0001	<0.0001	<0.0001	<0.0001	<0.0001	<0.0001	<0.0001
	A	0	7.570	<0.0001	<0.0001	<0.0001	<0.0001	<0.0001	<0.0001	<0.0001
	AB	B	1.639	0.1012	0.6070	0.4424	0.4727	0.4727	0.3565	0.4297
	AB	0	7.039	<0.0001	<0.0001	<0.0001	<0.0001	<0.0001	<0.0001	<0.0001
	B	0	4.826	<0.0001	<0.0001	<0.0001	<0.0001	<0.0001	<0.0001	<0.0001

Log-rank	A	AB	4.543	<0.0001	<0.0001	0.0001	<0.0001	<0.0001	<0.0001	<0.0001
	A	B	4.519	<0.0001	<0.0001	0.0001	<0.0001	<0.0001	<0.0001	<0.0001
	A	0	6.483	<0.0001	<0.0001	<0.0001	<0.0001	<0.0001	<0.0001	<0.0001
	AB	B	1.258	0.2084	1.0000	0.6634	0.7539	0.7539	0.5898	0.7716
	AB	0	5.924	<0.0001	<0.0001	<0.0001	<0.0001	<0.0001	<0.0001	<0.0001
	B	0	4.088	<0.0001	0.0003	0.0008	0.0003	0.0003	0.0003	<0.001

ModPeto	A	AB	5.103	<0.0001	<0.0001	<0.0001	<0.0001	<0.0001	<0.0001	<0.0001
	A	B	5.233	<0.0001	<0.0001	<0.0001	<0.0001	<0.0001	<0.0001	<0.0001
	A	0	7.570	<0.0001	<0.0001	<0.0001	<0.0001	<0.0001	<0.0001	<0.0001
	AB	B	1.638	0.1015	0.6089	0.4433	0.4738	0.4738	0.3573	0.4303
	AB	0	7.042	<0.0001	<0.0001	<0.0001	<0.0001	<0.0001	<0.0001	<0.0001
	B	0	4.829	<0.0001	<0.0001	<0.0001	<0.0001	<0.0001	<0.0001	<0.0001

Peto	A	AB	5.102	<0.0001	<0.0001	<0.0001	<0.0001	<0.0001	<0.0001	<0.0001
	A	B	5.231	<0.0001	<0.0001	<0.0001	<0.0001	<0.0001	<0.0001	<0.0001
	A	0	7.567	<0.0001	<0.0001	<0.0001	<0.0001	<0.0001	<0.0001	<0.0001
	AB	B	1.637	0.1016	0.6094	0.4435	0.4741	0.4741	0.3576	0.4312
	AB	0	7.040	<0.0001	<0.0001	<0.0001	<0.0001	<0.0001	<0.0001	<0.0001
	B	0	4.827	<0.0001	<0.0001	<0.0001	<0.0001	<0.0001	<0.0001	<0.0001

Tarone	A	AB	5.131	<0.0001	<0.0001	<0.0001	<0.0001	<0.0001	<0.0001	<0.0001
	A	B	5.153	<0.0001	<0.0001	<0.0001	<0.0001	<0.0001	<0.0001	<0.0001
	A	0	7.480	<0.0001	<0.0001	<0.0001	<0.0001	<0.0001	<0.0001	<0.0001
	AB	B	1.480	0.1388	0.8330	0.5338	0.5921	0.5921	0.4495	0.5594
	AB	0	6.887	<0.0001	<0.0001	<0.0001	<0.0001	<0.0001	<0.0001	<0.0001
	B	0	4.776	<0.0001	<0.0001	<0.0001	<0.0001	<0.0001	<0.0001	<0.0001

Wilcoxon	A	AB	5.100	<0.0001	<0.0001	<0.0001	<0.0001	<0.0001	<0.0001	<0.0001
	A	B	5.264	<0.0001	<0.0001	<0.0001	<0.0001	<0.0001	<0.0001	<0.0001
	A	0	7.598	<0.0001	<0.0001	<0.0001	<0.0001	<0.0001	<0.0001	<0.0001
	AB	B	1.688	0.0915	0.5488	0.4156	0.4376	0.4376	0.3301	0.3944
	AB	0	7.084	<0.0001	<0.0001	<0.0001	<0.0001	<0.0001	<0.0001	<0.0001
	B	0	4.840	<0.0001	<0.0001	<0.0001	<0.0001	<0.0001	<0.0001	<0.0001
